# Predicting plant attractiveness to pollinators with passive crowdsourcing

**DOI:** 10.1098/rsos.150677

**Published:** 2016-06-01

**Authors:** Christie A. Bahlai, Douglas A. Landis

**Affiliations:** Department of Entomology, Michigan State University, East Lansing, MI 48824, USA

**Keywords:** bee, data mining, nectar, pollination, search engine

## Abstract

Global concern regarding pollinator decline has intensified interest in enhancing pollinator resources in managed landscapes. These efforts frequently emphasize restoration or planting of flowering plants to provide pollen and nectar resources that are highly attractive to the desired pollinators. However, determining exactly which plant species should be used to enhance a landscape is difficult. Empirical screening of plants for such purposes is logistically daunting, but could be streamlined by crowdsourcing data to create lists of plants most probable to attract the desired pollinator taxa. People frequently photograph plants in bloom and the Internet has become a vast repository of such images. A proportion of these images also capture floral visitation by arthropods. Here, we test the hypothesis that the abundance of floral images containing identifiable pollinator and other beneficial insects is positively associated with the observed attractiveness of the same species in controlled field trials from previously published studies. We used Google Image searches to determine the correlation of pollinator visitation captured by photographs on the Internet relative to the attractiveness of the same species in common-garden field trials for 43 plant species. From the first 30 photographs, which successfully identified the plant, we recorded the number of *Apis* (managed honeybees), non-*Apis* (exclusively wild bees) and the number of bee-mimicking syrphid flies. We used these observations from search hits as well as bloom period (BP) as predictor variables in Generalized Linear Models (GLMs) for field-observed abundances of each of these groups. We found that non-*Apis* bees observed in controlled field trials were positively associated with observations of these taxa in Google Image searches (pseudo-*R*^2^ of 0.668). Syrphid fly observations in the field were also associated with the frequency they were observed in images, but this relationship was weak. *Apis* bee observations were not associated with Internet images, but were slightly associated with BP. Our results suggest that passively crowdsourced image data can potentially be a useful screening tool to identify candidate plants for pollinator habitat restoration efforts directed at wild bee conservation. Increasing our understanding of the attractiveness of a greater diversity of plants increases the potential for more rapid and efficient research in creating pollinator-supportive landscapes.

## 1. Introduction

Observations of pollinator decline have sparked global interest in managing for pollinator-supportive landscapes [1–6]. Climate change, habitat loss, pathogens and parasites, exposure to agrochemicals, loss of genetic diversity, malnutrition and in the case of managed bees, apicultural management have all been identified as potential (and interacting) drivers of this pollinator decline [2,7]. Owing to these factors, there is an increasing concern that managed honeybees will not be able to meet demands for agricultural pollination [1]. Wild bees function complementarily to managed bees and increased visitation by wild bees can enhance fruit set by a factor of 2 compared with a similar increase in visitation by honeybees [3]. Thus, developing landscape management and conservation tools that support a wide variety of pollinators is desirable to foster stability and productivity in agricultural systems [2].

Habitat manipulations can provide a variety of ecosystem service benefits in addition to pollination services, thus, non-crop habitat plantings have been an area of considerable research interest in recent years [3,8–16]. Agricultural landscape structure and composition strongly influence pollinators and pollination services [10,11,14]. Landscapes incorporating diverse floral resources can support a wide variety of pollinators, which in turn enhance pollination and yield in pollination-dependent crops occurring in these landscapes [10]. Although non-native plants can be used in habitat manipulations, and indeed, many non-native species can provide ample resources for pollinators [17], to provide maximum benefit to pollination and other ecosystem services, some habitat manipulations involving floral plantings emphasize native plant species [11,13,15]. Using locally adapted native plants has the potential to mitigate establishment and maintenance costs of floral plantings and because of shared evolutionary histories, better support native insects [13]. However, many studies rely on a limited set of plants that are well known to provide floral resources. Apiculturists have developed lists of ‘honey plants’ [18] and organizations, such as the Xerces Society, provide practical guidelines about implementing pollinator-friendly habitat management in farms [19]. Some plant species are included in these guidelines because they have been directly observed to attract pollinators or have undergone screening in common-garden experiments (e.g. [20]); however, much of the reasoning for including a plant in such lists is based on anecdotal evidence [21]. Use of these well-established lists limits the local customizability of habitat manipulation, because these recommendations tend to include broad geographical areas. However, performing a meaningful evaluation of the suitability of even a small portion of the native flora for all localities where floral plantings are desired is both expensive and logistically daunting. It is thus desirable to develop a screening tool to identify candidate plants for a variety of habitats.

Citizen science may be one tool that could help identify localized lists of plants for restoration. Citizen science is an increasingly popular methodology in conservation and restoration biology [22] and includes crowdsourcing, i.e. using the contributions of a large group of people to map trends or general perceptions. Ecologists and social scientists have sought to decentralize data collection through crowdsourcing methods for a variety of purposes [23,24]. For instance, insect ecologists have used citizen science approaches to develop an understanding of species distribution and habitat use and to detect rare species [25–27]. Most crowdsourced data are observational and can capture trends over broad spatial and temporal scales. Thus, these data function to complement or generate hypotheses for more directed, controlled experimental research [22].

Numerous crowdsourcing efforts seek to engage citizen scientists with specific research initiatives using common technologies, e.g. smartphone apps for collecting hydrologic or crop scouting data [28–31]. Others take a more general approach, such as iNaturalist (www.inaturalist.org), where community members are encouraged to submit photos of any and all organisms observed, tagged with relevant contextual information. Broader still, however, are observations that are produced as a matter of course of normal human Web use. Previous attempts to harness this sort of data, including the Google Flu Trends project, have been met with variable levels of success [32]; however, it is likely that the strength of relationships observed will vary dramatically with both the research area and the data collection medium. Billions of images exist on the Internet and have been indexed by various search engines [33]. These images may ‘passively’ capture scientifically relevant data: for example, a recent study used geotagged vacation photos shared on the popular photo sharing site Flickr (www.flickr.com) and user-reported profile data, to understand how lake quality affected how far a vacationer was willing to travel [34]. Similarly, floral photography is a common pastime among amateur and professional photographers. A Flickr search conducted in April 2014 yielded 92 103 photo sharing groups and over 23 million individual photographs tagged with the term ‘flower’ (C.A.B. 2014, personal observation). Many of these photos depict flowers occurring in natural or semi-natural habitats and in some cases, can capture relevant ecological information within the photo (such as insect visits) or within the photo caption (such as species identification). Google's Image search engine (www.google.com/imghp) provides a much broader database and additional search functionality. Image databases such as these represent a ‘passive’ crowdsourced data resource which has the potential to provide insights into ecological patterns and direct future experimental research efforts.

In this study, we use a ‘passive’ crowdsourced data resource to accelerate the search for pollinator-attractive plants. Specifically, we hypothesized that the abundance of Internet images of flowers with visiting insects may correspond to their attractiveness to insects under controlled experimental conditions. To test this hypothesis, we ask: ‘Is the frequency of observation of various pollinator taxa on plants in search engine results positively associated with the attractiveness of these plants under field conditions’?

## 2. Material and methods

### 2.1. Plant list and field observations

We used data produced by Tuell *et al.* [20] and Fiedler [35] to test the association between crowdsourced data and experimental results. In these previous studies, we and our co-workers have empirically measured the attractiveness of flowering plants to bee-mimic flower flies [15,35] and pollinators [20] in common-garden experiments using vacuum sampling (table 1). Specifically, these studies contrasted the attractiveness of five exotic plants that are widely recommended for their attractiveness to beneficial insects, to 43 species of perennial native plants [15]. Tuell & Fiedler [20] summarized their observations as number of *Apis* (honeybees) and non-*Apis* bees visiting each plant species at peak bloom. Because insect activity differed significantly over the course of the growing season, plants were grouped into three flowering categories: early, middle and late season blooming for analysis [20].

**Table 1.** Plant species used in image search experiment. Bloom period is for East Lansing, Michigan as reported in [15,20].

plant species		
Latin name	common name	bloom period
*Anemone canadensis*	meadow anemone	early
*Angelica atropurpurea*	angelica	early
*Aquilegia canadensis*	columbine	early
*Coreopsis lanceolata*	sand coreopsis	early
*Fragaria virginiana*	wild strawberry	early
*Geranium maculatum*	wild geranium	early
*Heracleum maximum*	wild cow-parsnip	early
*Heuchera americana*	alum root	early
*Hydrophyllum virginianum*	Virginia waterleaf	early
*Penstemon hirsutus*	hairy beardtongue	early
*Sambucus racemosa*	red elderberry	early
*Senecio obovatus*	round leaved ragwort	early
*Zizia aurea*	golden alexander	early
*Allium cernuum*	nodding wild onion	middle
*Amorpha canescens*	leadplant	middle
*Apocynum cannabinum*	Indian hemp	middle
*Asclepias incarnata*	swamp milkweed	middle
*Asclepias tuberosa*	butterfly weed	middle
*Ceanothus americanus*	New Jersey tea	middle
*Cephalanthus occidentalis*	buttonbush	middle
*Dasiphora fruticosa*	shrubby cinquefoil	middle
*Desmodium canadense*	showy tick trefoil	middle
*Oenothera biennis*	evening primrose	middle
*Ratibida pinnata*	yellow coneflower	middle
*Rosa setigera*	Michigan rose	middle
*Scrophularia marilandica*	late figwort	middle
*Spiraea alba*	meadowsweet	middle
*Verbena stricta*	hoary vervain	middle
*Veronicastrum virginicum*	Culver's root	middle
*Agastache nepetoides*	yellow giant hyssop	late
*Aster laevis*	smooth blue aster	late
*Cacalia atriplicifolia*	pale Indian plantain	late
*Eupatorium perfoliatum*	common boneset	late
*Helianthus strumosus*	paleleaf woodland sunflower	late
*Lespedeza hirta*	hairy bush-clover	late
*Liatris aspera*	rough blazing star	late
*Lobelia siphilitica*	great blue lobelia	late
*Monarda punctata*	horsemint	late
*Silphium perfoliatum*	cup plant	late
*Solidago riddellii*	Riddell's goldenrod	late
*Solidago speciosa*	showy goldenrod	late
*Symphyotrichum novae-angliae*	New England aster	late
*Vernonia missurica*	ironweed	late

Fiedler [35] used a similar vacuum sampling methodology, but instead focused on beneficial predators and parasitoids, including flower flies (Diptera: Syrphidae). Although syrphids have a predatory larval phase, they are nectar feeding as adults and many are important pollinators [36,37]. Additionally, adult syrphids are superficially similar to bees and often are mistaken for bees in photographs. Thus, we used the Tuell *et al.* [20] *Apis* and non-*Apis* bee observations and the Fiedler [35] syrphid observations by plant species as response variables.

### 2.2. Determining search engine and search terms

All searches were performed between December 2013 and April 2014 using the Google Chrome v. 33.0.x web browser. Search engines evaluated were Google Images (www.google.com/imghp) and Bing Images (www.bing.com/images/). To evaluate which search engine and search terms had best performance (i.e. yielded the most relevant results by returning images with the correct plant species and visible insects in the photo), we used the list of the five highly recommended exotic species from Fiedler & Landis [15] as these species are relatively common, frequently photographed and known to be attractive to beneficial insects. These species included *Vicia faba* (fava bean), *Fagopyrum esculentum* (buckwheat), *Coriandrum sativum* (coriander), *Lobularia maritime* (sweet alyssum), and *Anethum graveolens* (dill). Initial evaluation indicated that Latin names yielded more relevant search results (i.e. a greater number of photographs with correctly identified plants) than common names. Latin names were combined with the following search terms: ‘bee’, ‘beneficial insect’, ‘honeybee’ or ‘insect’. For the first 30 image results, which captured the correct plant species, the number of results where blooming flowers with insects present was recorded. If incorrectly identified plants or irrelevant images appeared, subsequent images were examined until a total of 30 images meeting these criteria were reached. The search term structure and search engine that yielded the most relevant results (Google Images, search term ‘[Plant Latin name] bee’) was used for all subsequent data collection (table 2).

**Table 2.** Number of search results meeting criteria of (i) correct plant species and (ii) insect present in photo from first 30 results of searches, one search for each combination of Latin plant name plus one of the additional search terms listed in the table, performed in two image search engines in searches conducted in early 2014. When fewer than 30 results were returned, the number of images searched for insect visitors is indicated in parentheses.

plant species			search term			
search engine	Latin name	common name	beneficial insect	insect	bee	honeybee
Bing						
	*Vicia faba*	fava bean	0 (7)	0	6	1 (17)
	*Fagopyrum esculentum*	buckwheat	0 (7)	5	8	3
	*Coriandrum sativum*	coriander	0 (19)	0	3	0 (27)
	*Lobularia maritima*	sweet alyssum	1	1	3	4
	*Anethum graveolens*	dill	0	0	1	0 (21)
	total		1	6	21	8
Google						
	*Vicia faba*	fava bean	2	3	11	8
	*Fagopyrum esculentum*	buckwheat	1	3	7	4
	*Coriandrum sativum*	coriander	1	4	2	2
	*Lobularia maritima*	sweet alyssum	1	1	2	2
	*Anethum graveolens*	dill	1	3	0	0
	total		6	14	22	16

### 2.3. Frequency of occurrence of pollinators in flower photos

**Figure 1.**
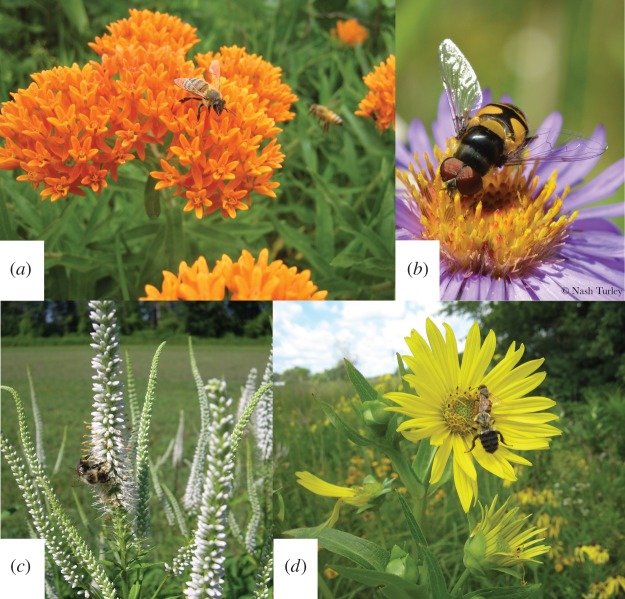
Examples of the types of images from Google Image searches for ‘[Plant Latin name] bee’ and meeting criteria for evaluation (see Material and methods). (*a*) *Apis* bee on *Asclepias tuberosa,* (*b*) syrphid fly on *Symphyotrichum novae-angliae*, (*c*) non-*Apis* bee on *Veronicastrum virginicum* and (*d*) one *Apis* bee (top), one non-*Apis* bee (bottom) on *Silphium perfoliatum*. Actual search result images are not reproduced here as many Google Image search results have copyright restrictions. Photos (*a*), (*c*) and (*d*) by D. Landis. Photo (*b*) by Nash Turley (www.nashturley.org/), reproduced with permission.

Using Google Image search, we conducted searches for ‘[Plant Latin name] bee’ using the list of native Michigan plants that were used in our group's common-garden studies (table 1; [15,20,35]. Images were evaluated sequentially, in the order they appeared in the search results. Images were evaluated by the following criteria: (i) each search procedure received one tally for each image containing the correct plant species, shown in bloom, and with sufficient image quality such that target insect taxa could be reliably identified (i.e. the image was not blurry and the inflorescence was clearly visible), (ii) in the set of images where the previous condition was met, the number of images where *Apis*, non-*Apis* bees and syrphid flies were visible were tallied and recorded (figure 1). For each search procedure, photos were sequentially evaluated until 30 images meeting criterion 1 were evaluated or until 200 images were examined. The number of images evaluated for each search was recorded. Duplicate images and differently cropped shots of previously counted images were excluded from evaluation and not counted towards the total images searched.

### 2.4. Analysis

Search data (*S*) were compared with data from the field studies [15,20,35]. Because some searches did not have 30 images meeting the criteria described above, search results were scaled for lower search success rates by multiplying the number of images where a given taxon was observed by 30 and dividing by the number of images meeting the criteria in that category. Then, a model selection approach was used to determine which parameters were in the best model to predict field observations (*O*) for a given pollinator taxon (all bees, *Apis* bees, non-*Apis* bees and syrphids). Because net bee abundance varied dramatically by bloom period (BP) (as defined for Michigan native plants in Tuell *et al.* [20]), this variable (BP) was also included as in the model selection procedure. The field observations took the form of counts, so models with Poisson or negative binomial error structure are most appropriate [38]. As models fit reasonably well (i.e. the ratio of residual deviance to residual degrees of freedom less than 1 for all models), Poisson structures were used for all analyses. The global model, a GLM took the form

2.1 ln(O)∼S+BP+S×BP.

For each pollinator group-based model set, variables were dropped singly from the global model to determine the simplest model with the best performance. Akaike's Information Criterion (AIC) [39] was used to rank models. If two models had equivalent performance (i.e. produced AIC values that were within two units of each other), the model with the fewest parameters was selected as the best model. All analyses were performed in R v. 3.0.3 [40]. Figures were generated with ggplot2 [41]. An *α *= 0.05 was used to determine statistical significance, where appropriate.

## 3. Results

### 3.1. Frequency of occurrence of pollinators in flower photos

We examined a total of 3281 images resulting from 43 searches. An average of 28.8 images (range 5–30 images) for each plant species met our criterion for image quality and an average of 8.2 images (range 0–27) per plant species depicted insects. *Apis* bees were visible in 5.6% of images meeting criteria and non-*Apis* bees and syrphids were present in 17.5% and 1.9% of images, respectively.

### 3.2. Relationship to field data

**Figure 2.**
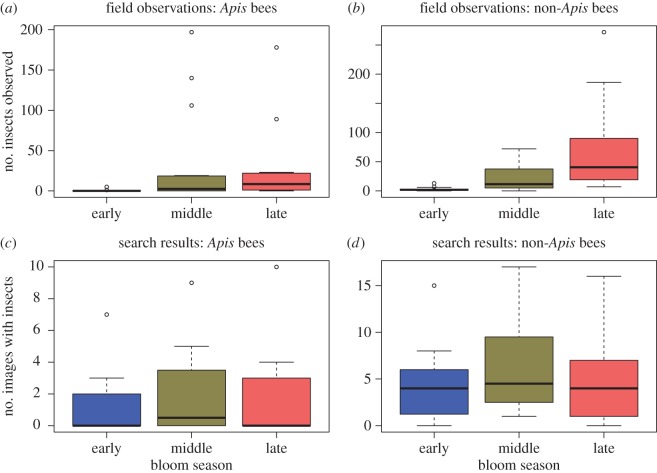
Boxplots of field and Google image observations of insect visitations by bloom season. Field observations (*a* and *b*) are based on data collected by Tuell *et al.* [20], search results (*c* and *d*) display frequency of insects appearing in images meeting criteria (see Material and methods).

**Figure 3.**
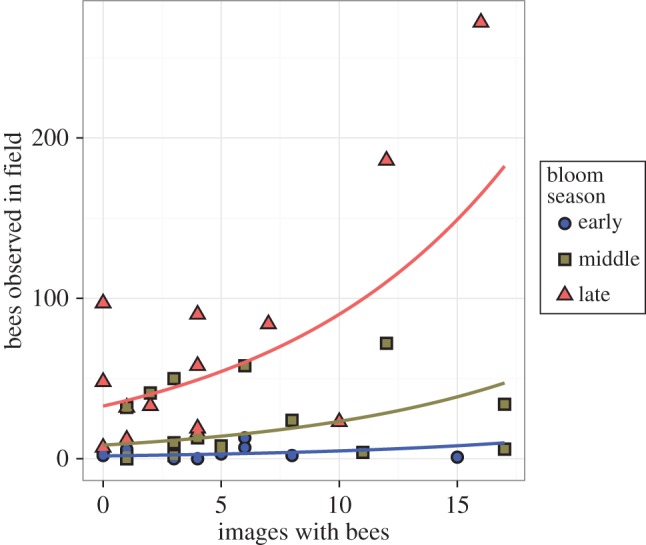
Best model for non-*Apis* bees, depicting the number of non-*Apis* bees by plant species observed by Tuell *et al.* [20] as a function of BP and the number of observations of non-*Apis* bees on that plant species in Google Image searches. Each point represents observations for a single plant species.

Model selection favoured the inclusion of image search results in models for field observations of all bees, non-*Apis* bees and syrphids, and all these regressions produced positive regression coefficients (table 3). The best model for *Apis* bees only included BP. BP was also included in the best models for non-*Apis* bees and all bees, but not in the model for syrphids. Only the models for all bees and non-*Apis* bees produced statistically significant regression parameters, although effects in the ‘all bee’ model was largely due to responses of non-*Apis* bees, as *Apis* bees represented a minority of those observed. Observations of both *Apis* and non-*Apis* bees were more variable by BP in search result data than in field-collected data (figure 2). Non-*Apis* bee field observations had the strongest relationship with search result data (figure 3).

**Table 3.** Summary of model selection results. Variables included in best models as determined by AIC are indicated with a Y. Pseudo-*R*^2^ values given are the Cragg & Uhler's [42] pseudo-*R*^2^ for the best model. All regressions used models with Poisson error structure.

model	number of images (*S*)	slope (±s.e.)	bloom period (BP)	interaction *S* × BP	pseudo-*R*^2^
all bees^a^	Y	0.05 ± 0.02	Y	—	0.594
*Apis* bees	—	—	Y	—	0.303
non-*Apis* bees^a^	Y	0.10 ± 0.04	Y	—	0.668
syrphids	Y	0.08 ± 0.72	—	—	0.003

^a^Models with statistically significant regression parameters at *α *= 0.05.

## 4. Discussion

We detected positive associations between the frequency that non-*Apis* bees were photographed on a given plant and its relative attractiveness to non-*Apis* bees in controlled field trials (figure 3). To a lesser and much more variable extent, a similar positive association was observed for total bees and syrphid flies (table 3). We did not observe this relationship for *Apis* bees. The reason for this strong association observed for non-*Apis* bees compared with other taxa may, at least in part, be due to sample size effects: non-*Apis* bees were observed nearly twice as often as *Apis* bees in the field and more than 10 times as often as syrphid flies [20,35], thus relationships may not be consistent enough to be statistically detectable. However, model selection suggested that unlike non-*Apis* bees and syrphids, *Apis* bees were only associated with BP of flowers (table 3 and figure 2). The model for total bees, defined as the sum of non-*Apis* and *Apis* bees, although statistically significant, had a substantially lowered strength of effect (i.e. slope) between the number of bees observed in the field versus their frequency of observation in photos. This result suggests that conflicting responses essentially masked the strong association observed in the non-*Apis* bee model and highlights the importance of striking a balance between taxonomic resolution and available sample size.

Both honeybee behaviour and human manipulation of their colonies may play a role in the differentiation of patterns we observed between *Apis* and non-*Apis* bees. Model selection favoured a model containing only BP to predict *Apis* bee abundance, suggesting that seasonality, potentially related to cropping practices and not the specific attributes of a particular flower species, is the primary factor driving honeybee visitation, at least in Michigan field trials. Honeybees are generalist foragers, which are moved from crop to crop, as pollination needs dictate [2,43]. This management practice adds an element of unpredictability to their foraging patterns: colonies of *Apis* bees are physically moved throughout the season, thus their use of plants adjacent to croplands would be a function of colony placement and attractiveness of their target crop. This seasonality effect would vary with region, crop and local apicultural practice, and thus could obscure patterns in image search results, which draw from a global range. The social behaviour of *Apis* bees also influences the foraging behaviour of the colony. Scout bees inform nest-mates of the direction and distance to flowering resources [44] and honeybees tend to have high fidelity to specific resources where they have previously found significant reward [45]. In combination, these behaviours may influence bee abundance at floral resources that are less abundant in the landscape.

The results of this study have potential application in the development of locally targeted pollinator enhancement habitats, particularly those that emphasize supporting wild pollinator populations. Locally targeted pollinator plantings, particularly those emphasizing native plants, are desirable from a wide variety of perspectives. In addition to supporting restoration of native plant diversity, habitat enhancements emphasizing native plants help to restore local biodiversity. Floral resources can increase local biodiversity by supporting specialist insects that may be endemic to the area and plants can provide non-floral resources year-round, such as nesting and overwintering sites [11,46]. Furthermore, using native plants that tolerate local environmental conditions can help to lower establishment and maintenance costs of these habitats [13].

Although we did not observe any generalizable trends in *Apis* bee plant preferences, honeybees also benefit from well-designed pollinator habitats in landscapes. Honeybees have high energy requirements and habitats with an abundance of nectar available season-long are better able to support larger honeybee colonies [47,48]. Even if honeybee colonies are being moved through the landscape for crop pollination, honeybees can and do forage within wild plant communities embedded in agricultural matrices [20,48]. Locally targeted pollinator enhancement habitats can support greater communities of natural enemies, as well as supporting conservation biological control and potentially mitigating pesticide risk [11–15,49]. Our methodology serves as a complement to strategies already in place for developing pollinator habitats and helps to refine efforts for creating locally adapted plant communities. Using our methodology, plant lists with a particular set of attributes (i.e. adapted to a particular soil type, endemic to a specific region) can be evaluated for further screening under field conditions.

Crowdsourcing data allow us to use a collective intelligence which can outperform individual studies or experts [50]. Crowdsourcing usually capitalizes on the intent of the participants to produce data for a specific purpose, but incidental observations of casual Web users, mined for patterns, can be regarded as a ‘passive’ crowdsourcing approach. Using passively crowdsourced data and the methodology outlined in this study may have applications in other systems. Internet images can represent a random sample of events and as we have shown, at least for certain interactions, the frequency with which an event is observed in Internet photos corresponds to the frequency of events occurring in the field, under controlled conditions. Yet, it is important that findings based on this methodology be ‘ground-truthed’. Not all patterns will be captured because of localized variability. If the geographical range of a particular interaction is wide and patterns in the interactions vary over the range, this decreases the likelihood that a usable trend will be detected. Geographical biases affecting data quality would also include cultural and economic factors (i.e. the availability of photographic equipment and Internet access in a given region, the local cultural precedence for collecting images of organisms and sharing them on the Internet, the time of year people are most likely to use leisure time to photograph insects or flowers). Additionally, citizen scientists are more likely to document rare events [27], possibly due to cognitive biases associated with the recall of unusual occurrences [22,51]. Comparing the results of searches to experimental results is essential to develop an understanding of which interactions are captured in images and which are not. Yet, as we have shown, our methodology has the potential to have application in capturing a subset of ecological interactions with potential implications in management.

## Data accessibility

R code used for analysis is available in an online repository at https://github.com/cbahlai/Bee_images/blob/master/Bee_images.R. Data used in analysis are available online at http://dx.doi.org/10.6084/m9.figshare.1053150.
